# Note on the Refraction of Seven Hundred Men

**Published:** 1896-08

**Authors:** Richard H. Satterlee

**Affiliations:** Buffalo, N. Y. 189 Delaware Avenue


### NOTE ON THE REFRACTION OF SEVEN HUNDRED MEN.

By RICHARD H. SATTERLEE, M. D., Buffalo, N. Y.

IN THE EXAMINATION of 700 men during the past ten months for employment on a railway, 90 per cent. were farmers, with now and then a laborer, carpenter, soldier, mason, student, clerk, teacher, telegrapher, lawyer, actor, detective, plumber or sailor.

The standard of vision required was 20-20 in each eye. Of the 700 only twenty-three fell short of the standard. The refraction was as follows:

| | | |
|---|---|---|
| *Hypermetropia* | less than one diopter in both eyes | 199 |
| | one diopter or more in both eyes | 490 |
| | more in right eye than left eye | 60 |
| | more in left eye than right eye | 9 |
| | highest degree of | 8 dioptries. |
| *Myopia* | | 3 |
| *Astigmatism* | less than one diopter | 599 |
| | axis other than 90 degrees | 57 |
| | highest degree of | 6 dioptries. |
| | mixed (h. and m.) | 4 |
| *Anisometropia* | | 4 |
| *Visual acuteness* | slightly better in right eye | 257 |
| | slightly better in left eye | 34 |

*Muscular defects.* A number had a deviation from what is considered the normal standard, but only three showed any marked defect.

189 Delaware Avenue.

---

Milk Filtered Through Cotton.—Dr. Seibert, of New York, has made a number of experiments with milk filtered through cotton. He finds that while the greater part of the germs are eliminated no cream is lost. Plain milk showed from thirty-eight hundred to two hundred thousand germs, while the same milk filtered showed about one-fourth that number. If this is true, filtration is about as good as sterilisation, and has an advantage in that sterilised milk is not so good a food as plain milk.—*Canadian Practitioner.*

